# Supplementary material for: Comparative Analysis of HPV16 Variants in the Untranslated Regulatory Region, L1, and E6 Genes among Vaccinated and Unvaccinated Young Women: Assessing Vaccine Efficacy and Viral Diversity
Source: Viruses. 2024 Aug 29;16(9):1381. doi: 10.3390/v16091381 (PMC11435937; doi:10.3390/v16091381)
Supplement: Supplementary file 1 [file viruses-16-01381-s001.zip › viruses-3153088-supplementary.pdf]

Table S1. Overview of L1 and URR/E6 pre-PCR and nested-PCR primers sequences. Start positions are according to HPV16 reference sequence K02718.1.

Region	Forward		Reverse	
	Start	Sequence (5'-3')	Start	Sequence (5'-3')
L1-pre	5432	CAATTCCTTTTGGTGGTGCAT	7289	GACACAATAGTTACACAAGCAT
L1-nst -1	5597	TGTTACGAAAACGACGTAAACG	5978	GATGGCCACTAATGCCCACA
L1-nst -2	5919	ATACACAGCGGCTGGTT	6353	AGCTGTCGCCATATGGT
L1-nst -3	6284	AACAAAAGTGAAGTTCCACTGG	6664	AGATATGGCAGCACATAATGAC
L1-nst -4	6600	TGGCATTTGTTGGGGTAAC	7205	TACAAGCACATACAAGCACAT
URR/E6-pre	6934	ACCTCCAGCACCTAAAGAAGATC	692	TAATGGGCTCTGTCCGGTTCTG
URR/E6-nst -1	7090	ACGAAAAGCTACACCCACCACCTCA	7429	TGAAGCTACAAAATGGCCGCTGGC
URR/E6-nst -2	7306	GTCATGCAACATAAATAAAC	7717	CAAGCCAAAAATATGTGC
URR/E6-nst -3	7337	CAACACCTACTAATTGTGTTGTGGT	7665	GTAAGGCGTTGGCGCATAGTGATTT
URR/E6-nst -4	7585	GCTTGCCAACCATTCCATTGTTTTT	110	GGTCGCTCCTGTGGGTCCTG
URR/E6-nst -5	50	ACCGAAACCGGTTAGTATAAAAGCA	415	GGACACAGTGGCTTTTGACAGTTA

Table S2. Overview of the pre-PCR and nested-PCR primers used for the amplification of the L1 and URR/E6 regions.

	URR/E6			L1		
Pre-PCR	°C	Duration	Cycles	°C	Duration	Cycles
Denaturation	95	5min	1x	95	5min	1x
Denaturation	95	30sec		95	30sec	
Annealing	55	30sec	45x	50	30sec	45x
Elongation	72	1.5 min		72	2min	
Final Elongaion	72	10min	1x	72	10min	1x
Cooling	4	infinite	1x	4	infinite	1x
nested-PCR	°C	Duration	Cycles	°C	Duration	Cycles
Denaturation	95	5min	1x	95	5min	1x
Denaturation	95	30sec		95	30sec	
Annealing	55	30sec	45x	50	30sec	45x
Elongation	72	30sec		72	45sec	
Final Elongaion	72	10min	1x	72	10min	1x
Cooling	4	infinite	1x	4	infinite	1x

Table S3. Overview of the number of SNPs (and frequency, %) found across the L1, URR and E6 regions of HPV16 among vaccinated and unvaccinated women.

**SNPs found across the L1 gene**										
Position L1	T21C	G60A	T189C	A195C	C226T	T273C	C289A	T324C	G345A	C527A
Position whole genome	T5659C	G5698A	T5827C	A5833C	C5864T	T5911C	C5927A	T5962C	G5983A	C6165A
Vaccinated women (n=16)	0 (0)	0 (0)	1 (6.3)	0 (0)	0 (0)	0 (0)	0 (0)	1 (6.3)	0 (0)	0 (0)
Unvaccinated women (n=25)	1 (4)	3 (12)	1 (4)	1 (4)	3 (12)	3 (12)	1 (4)	1 (4)	1 (4)	3 (12)
p-value	1	0.27	1	1	0.27	0.27	1	1	1	0.27
Position L1	T549G	T609C	A678G	A717C	A753G	A796G	A867C	C921T	T930A	T1050G
Position whole genome	T6187G	T6247C	A6316G	A6355C	A6391G	A6434G	A6505C	C6559T	T6568A	T6688G
Vaccinated women (n=16)	0 (0)	0 (0)	0 (0)	0 (0)	1 (6.3)	12 (75)	0 (0)	0 (0)	0 (0)	0 (0)
Unvaccinated women (n=25)	1 (4)	3 (12)	3 (12)	1 (4)	3 (12)	19 (76)	1 (4)	3 (12)	1 (4)	1 (4)
p-value	1	0.27	0.27	1	1	1	1	0.27	1	1
Position L1	A1057C	G1083A	T1128C	T1186C	C1216T	T1224C	C1227T	A1290C	C1332T	G1356A
Position whole genome	A6695C	G6721A	T6766C	T6824C	C6854T	T6862C	C6865T	A6928C	C6970T	G6994A
Vaccinated women (n=16)	0 (0)	0 (0)	0 (0)	0 (0)	0 (0)	1 (6.3)	0 (0)	1 (6.3)	0 (0)	0 (0)
Unvaccinated women (n=25)	2 (8)	3 (12)	2 (8)	1 (4)	3 (12)	0 (0)	2 (8)	0 (0)	3 (12)	3 (12)
p-value	0.51	0.27	0.51	1	0.27	0.39	0.51	0.39	0.27	0.27
Position L1	A1361G	G1422A	G1422T	A1449G						
Position whole genome	A6999G	G7060A	G7060T	A7087G						
Vaccinated women (n=16)	1 (6.3)	0 (0)	0 (0)	1 (6.3)						
Unvaccinated women (n=25)	1 (4)	1 (4)	3 (12)	0 (0)						
p-value	1	1	0.27	0.39						
**SNPs found across the URR region**										
Position URR	A18C	G37T	A39G	A76G	A77C	C154G	A160C	T172G	G203A	G204A
Position whole genome	A7174C	G7193T	A7195G	A7232G	A7233C	C7310G	A7316C	T7328G	G7359A	G7360A
Vaccinated women (n=16)	1 (6.3)	12 (75)	0 (0)	0 (0)	0 (0)	1 (6.3)	0 (0)	1 (6.3)	0 (0)	0 (0)
Unvaccinated women (n=25)	0 (0)	18 (72)	1 (4)	1 (4)	3 (12)	0 (0)	2 (8)	0 (0)	2 (8)	2 (8)
p-value	0.39	1	1	1	0.27	0.39	0.51	0.39	0.51	0.51
Position URR	C223G	T237A	C238T	G273T	G280A	T285G	T294C	T313A	A329C	G333A
Position whole genome	C7379G	T7393A	C7394T	G7429T	G7436A	T7441G	T7450C	T7469A	A7485C	G7489A
Vaccinated women (n=16)	0 (0)	1 (6.3)	1 (6.3)	0 (0)	1 (6.3)	0 (0)	1 (6.3)	0 (0)	0 (0)	0 (0)
Unvaccinated women (n=25)	1 (4)	0 (0)	0 (0)	1 (4)	0 (0)	2 (8)	2 (8)	1 (4)	2 (8)	3 (12)
p-value	1	0.39	0.39	1	0.39	0.51	1	1	0.51	0.27
Position URR	T340C	A351G	G365A	G396A	C434T	A506C	C513T	C533A	T558A	A573C
Position whole genome	T7496C	A7507G	G7521A	G7552A	C7590T	A7662C	C7669T	C7689A	T7714A	A7729C
Vaccinated women (n=16)	1 (6.3)	0 (0)	12 (75)	0 (0)	1 (6.3)	0 (0)	0 (0)	0 (0)	0 (0)	0 (0)
Unvaccinated women (n=25)	3 (12)	1 (4)	19 (76)	1 (4)	0 (0)	1 (4)	2 (8)	3 (12)	1 (4)	2 (8)
p-value	1	1	1	1	0.39	1	0.51	0.27	1	0.51
Position URR	C608T	C628T	C630T	C636G	G678T	G712A	C720A	A721C	T762C	C781T
Position whole genome	C7764T	C7784T	C7786T	C7792G	G7834T	G7868A	C7876A	A7877C	T7918C	C7937T
Vaccinated women (n=16)	0 (0)	0 (0)	0 (0)	1 (6.3)	0 (0)	3 (18.8)	0 (0)	0 (0)	1 (6.3)	0 (0)
Unvaccinated women (n=25)	3 (12)	2 (8)	3 (12)	0 (0)	3 (12)	0 (0)	1 (4)	1 (4)	0 (0)	1 (4)
p-value	0.27	0.51	0.27	0.39	0.27	0.05	1	1	0.39	1
Position URR	C823T	A833C	C849T							
Position whole genome	C7979T	A7989C	C8005T							
Vaccinated women (n=16)	1 (6.3)	0 (0)	0 (0)							
Unvaccinated women (n=25)	0 (0)	1 (4)	1 (4)							
p-value	0.39	1	1							
**SNPs found across the E6 gene**										
Position E6	T6C	A28G	G29C	C40G	G42T	T148A	T183A	A186G	C232T	T247G
Position whole genome	T109C	A131G	G132C	C143G	G145T	T251A	T286A	A289G	C335T	T350G
Vaccinated women (n=16)	1 (6.3)	1 (6.3)	0 (0)	0 (0)	0 (0)	1 (6.3)	0 (0)	0 (0)	0 (0)	8 (50)
Unvaccinated women (n=25)	2 (8)	0 (0)	1 (4)	1 (4)	3 (12)	0 (0)	3 (12)	3 (12)	3 (12)	13 (52)
p-value	1	0.39	1	1	0.27	0.39	0.27	0.27	0.27	1

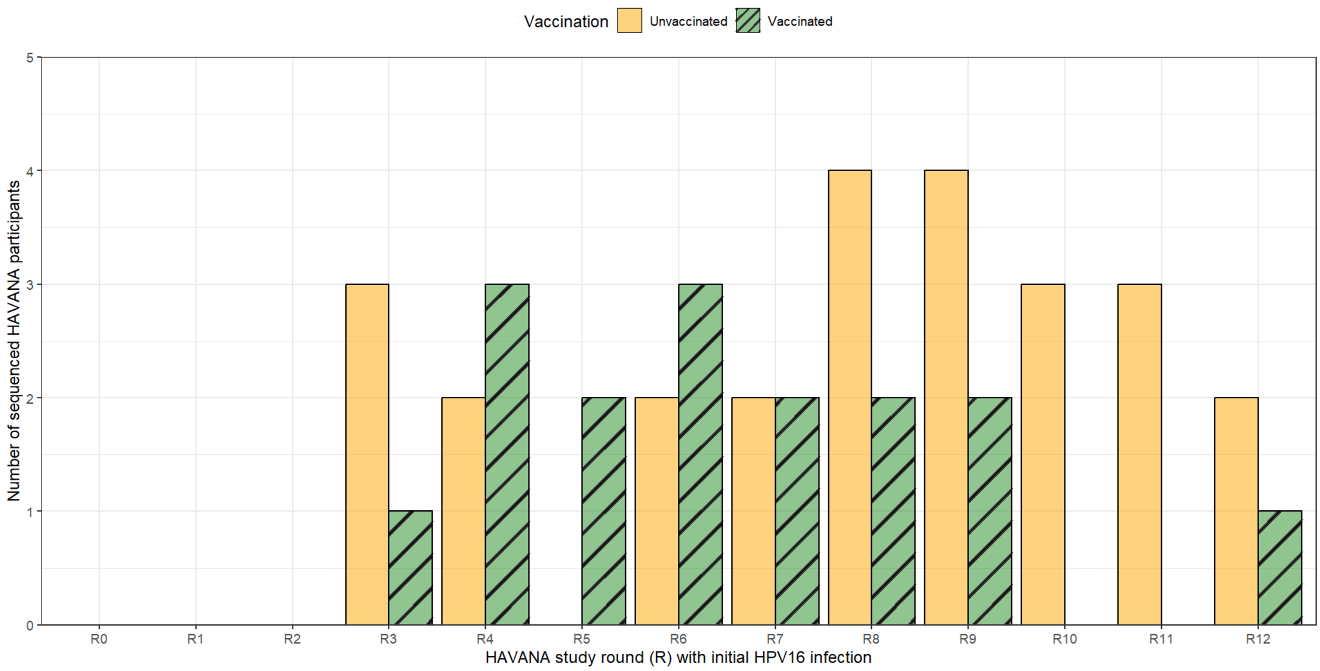

Figure S1. The time in study rounds from the beginning of the HAVANA study, at enrollment, to initial HPV16 infection and the number of sequenced vaccinated women (green striped bar) and sequenced unvaccinated women (yellow bar).

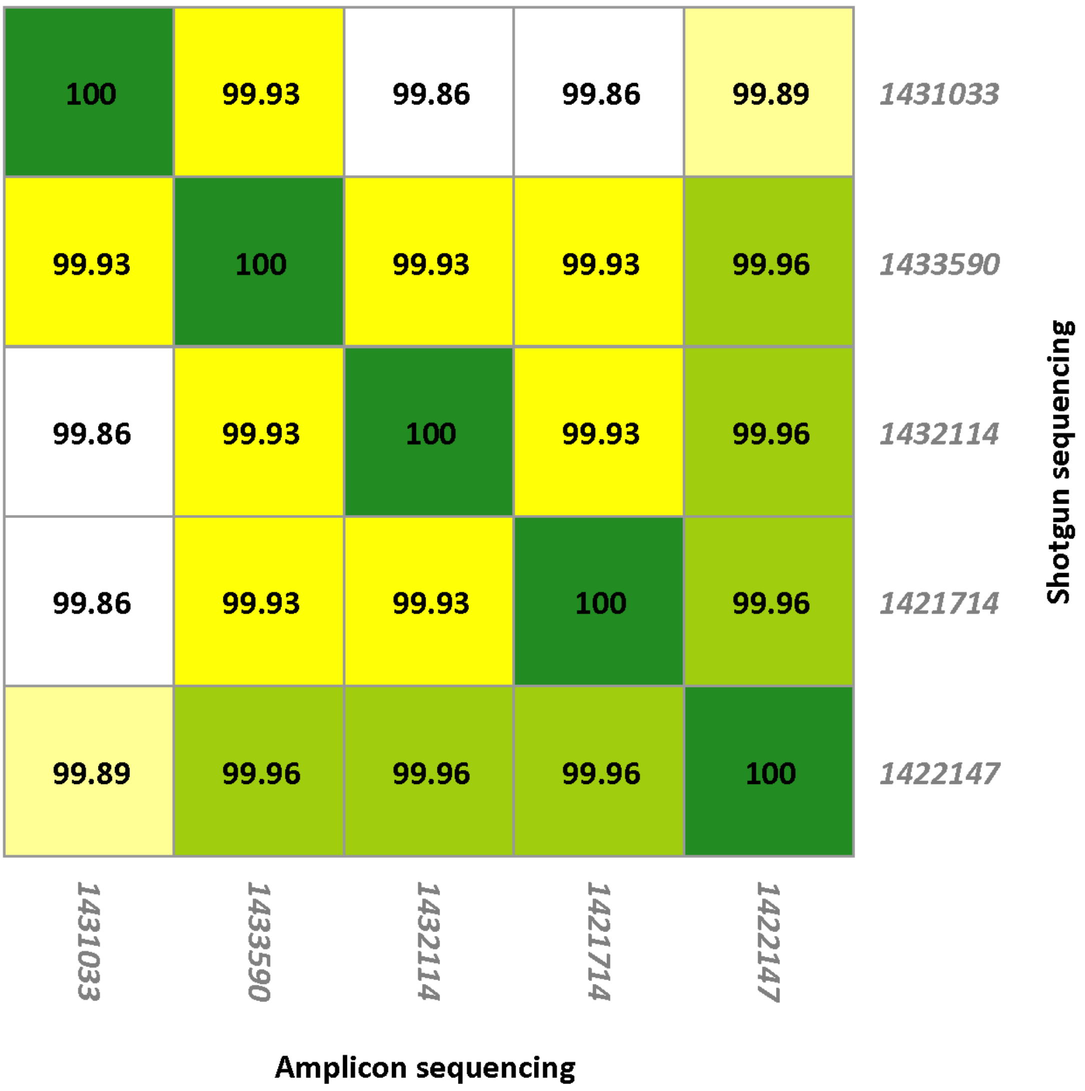

Figure S2. Heatmap which shows the nucleotide similarity (%) between shotgun-generated (y-axis) and amplicon-generated (x-axis) sequences from the subset of 5 samples. A similarity of 100% is given the color 'dark green'.

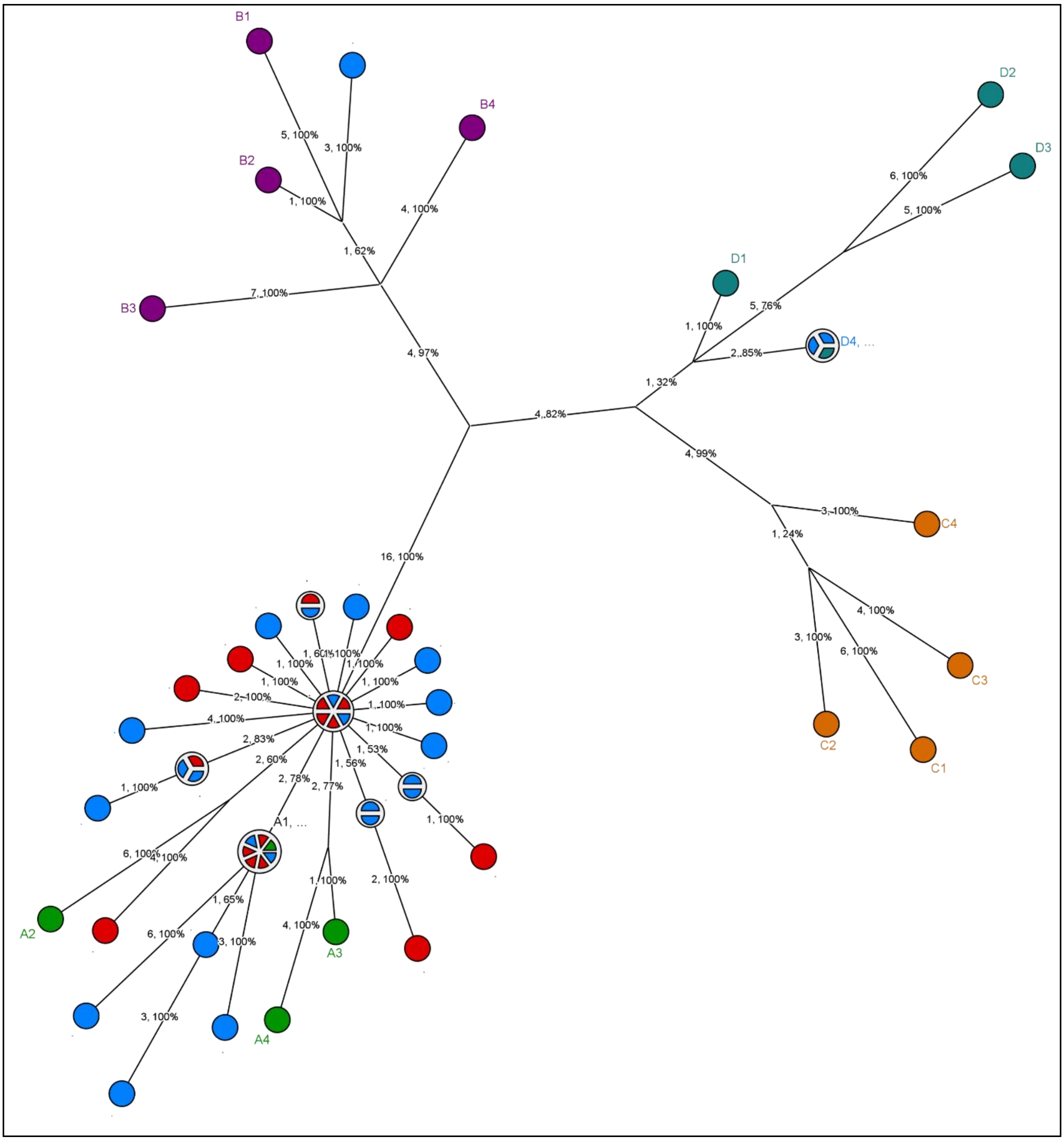

Figure S3. Maximum parsimony tree of the HPV16 L1, URR and E6 sequences from vaccinated (red, n=16) and unvaccinated women (blue, n=25), together with reference sequences from lineage A (green, n=4), lineage B (purple, n=4), lineage C (yellow, n=4) and lineage D (turquoise, n=4). Each circle represents a specific variant and along the branches the number before the comma represents the number of nucleotide differences and the percentage behind the comma the bootstrap value.

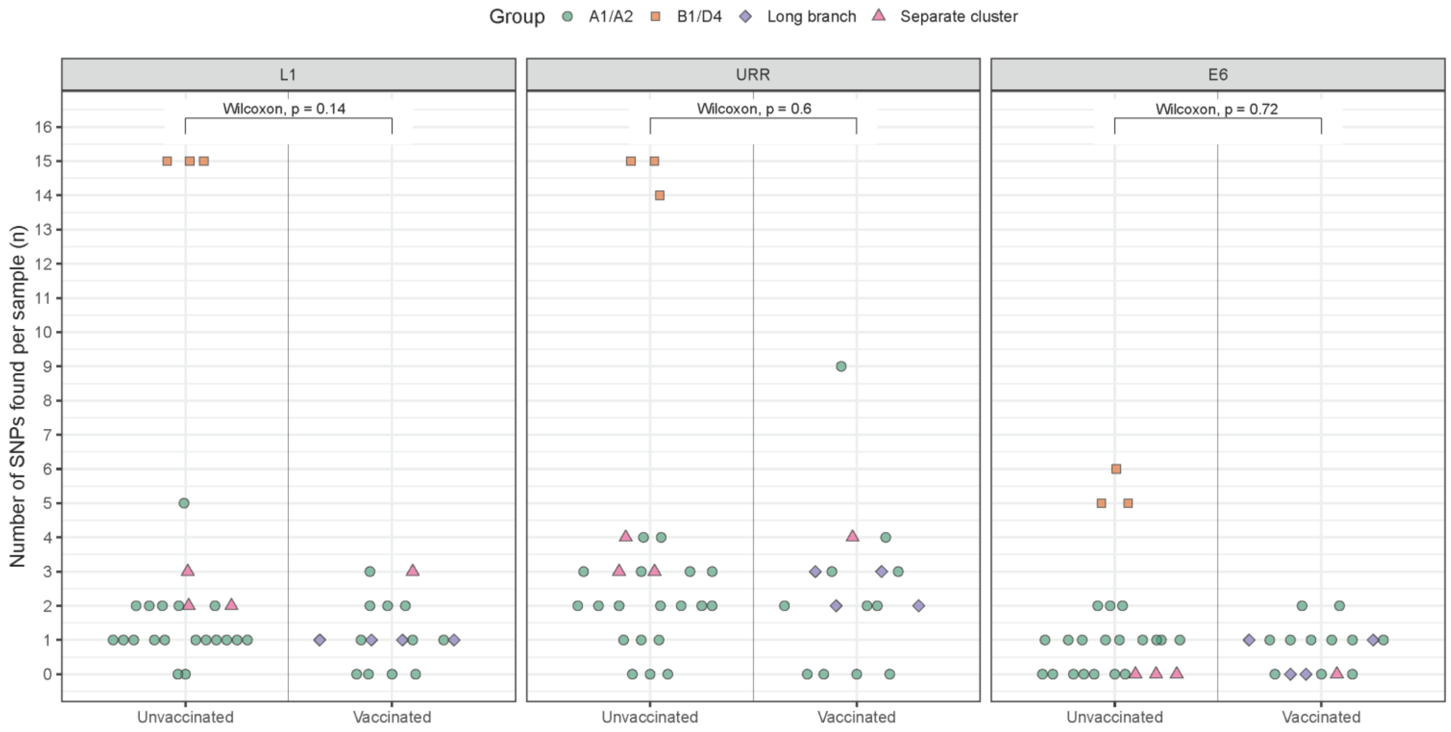

Figure S*4*. The number of SNPs found per variant among vaccinated and unvaccinated women in the L1 (left panel), URR (middle panel) and E6 (right panel). The variants are categorized as follows: A1/A2 (turquoise circles), B1/D4 (orange squares), the variants within lineage A with a long branch (purple diamonds) and the separate cluster within lineage A (pink *triangles).*
